# Supplementary material for: Costs and cost-effectiveness of cervical cancer screening strategies in women living with HIV in Burkina Faso: The HPV in Africa Research Partnership (HARP) study
Source: PLoS One. 2021 Mar 25;16(3):e0248832. doi: 10.1371/journal.pone.0248832 (PMC7993811; doi:10.1371/journal.pone.0248832)
Supplement: S1 Table — (DOCX) [file pone.0248832.s001.docx]

**Supplementary table 1. Breakdown of tests by cost component in 2019 United States Dollars.**

| **Test** | **Personnel** | **Training** | **Consumables** | **Capital** | **Overheads** | **Total** |
| --- | --- | --- | --- | --- | --- | --- |
| VIA | $ 0.5 |  | $ 1.9 | $ 0.2 | $ 0.6 | $ 3.2 |
| VIA/VILI | $ 0.5 |  | $ 2.1 | $ 0.2 | $ 0.7 | $ 3.6 |
| *care*HPV | $ 0.8 | $ 3.7 | $ 16.9 | $ 0.5 | $ 5.5 | $ 27.3 |
| Cytology | $ 3.8 |  | $ 15.0 | $ 1.0 | $ 5.0 | $ 24.8 |
| Colposcopy | $ 1.9 |  | $ 2.5 | $ 0.9 | $ 1.3 | $ 6.6 |
| Single biopsy including colposcopy | $ 5.2 |  | $ 13.3 | $ 7.9 | $ 6.6 | $ 33.0 |
| Four-quadrant biopsy including colposcopy | $ 8.8 |  | $ 21.6 | $ 7.9 | $ 9.6 | $ 47.9 |
