# Supplementary material for: Costs and cost-effectiveness of cervical cancer screening strategies in women living with HIV in Burkina Faso: The HPV in Africa Research Partnership (HARP) study
Source: PLoS One. 2021 Mar 25;16(3):e0248832. doi: 10.1371/journal.pone.0248832 (PMC7993811; doi:10.1371/journal.pone.0248832)
Supplement: S2 Table — For the non-dominated screening strategies, the sensitivity was decreased and increased by 20%. (DOCX) [file pone.0248832.s002.docx]

**Supplementary table 2. Results of the one-way sensitivity analysis on the sensitivity of screening strategies in 2019 United States Dollars.** The sensitivity of screening strategies was decreased and increased by 20% for those that were non-dominated.

| Parameter | ICER | |
| --- | --- | --- |
|  | VIA/VILI | *care*HPV |
| VIA low sensitivity (35%) | $28 | $814 |
| VIA high sensitivity (53%) | $161 | $814 |
| VIA/VILI low sensitivity (45%) | $483 | $638 |
| VIA/VILI high sensitivity (68%) | $25 | $1,126 |
| *care*HPV low sensitivity (78%) | $48 | $1,557 |
| *care*HPV high sensitivity (100%)^a^ | $48 | $756 |

*care*HPV, HPV DNA test; ICER, incremental cost-effectiveness ratio; VIA, visual inspection with acetic acid; VIA/VILI, combined visual inspection with Lugol’s iodine.

^a^ This is the maximum sensivity, which is less than a 20% increase from the base case analysis.
