# Supplementary material for: Costs and cost-effectiveness of cervical cancer screening strategies in women living with HIV in Burkina Faso: The HPV in Africa Research Partnership (HARP) study
Source: PLoS One. 2021 Mar 25;16(3):e0248832. doi: 10.1371/journal.pone.0248832 (PMC7993811; doi:10.1371/journal.pone.0248832)
Supplement: Attachment — Submitted filename: Response to reviewers.docx [file pone.0248832.s004.docx]

**1. Thank you for the updates to the manuscript and particularly the limitations/discussion section on the challenges associated with interpreting cost per case detected, since conclusions and recommendations may be different when evaluating cervical cancer prevention as the end point. Please consider including this in the abstract conclusions as well, to support appropriate reader (and potential end-user) interpretation of cost per case detected results.**

Thanks very much for this and the following helpful suggestions to further improve the manuscript. We have amended the abstract conclusions to (lines 59-64):

“Depending on the willingness to pay for the detection of a case of cervical cancer, decision makers in Burkina Faso can consider a variety of cervical cancer screening strategies for WLHIV. While careHPV is more costly, it has the potential to be cost-effective depending on the willingness to pay threshold. Future research should explore the lifetime costs and benefits of cervical cancer screening to enable comparisons with interventions for other diseases.” **2. Table 2. The additional details in the manuscript and Table 2 (PPV, true CIN2+ cases detected and colposcopy) are helpful. However, the information on colposcopy in Table 2 is a bit laborious for the reader to interpret as presented (some percents, some #s). Consider including both Ns and %s for the columns of women receiving colpo and women without CIN2 receiving colpo, or clarify interpretation of these columns with a table footnote to improve clarity.**

We have added these to Table 2 and amended errors in the numbers for those receiving colpo without CIN2+, which added to the difficulty in interpreting these numbers.

**3. Thank you for noting that a sensitivity analysis for VIA/VILI and cytology were conducted. The author response suggests this is included in Figure 2, but Figure 2 appears to be reporting the univariate analysis for careHPV. Please clarify where this information can be found.**

Apologies that this update didn’t make it into the last submission. Figure 2 now includes VIA/VILI and cytology.

**4. Time horizon: thanks for adding the time horizon. Suggest explicitly noting whether costs/outcomes were discounted (assuming not because the time horizon was less than one year).**

This has been added to line 253: “Costs and outcomes were not discounted.”

**5. Line 446-448: "While our analysis indicates that it is cost-effective compared to VIA alone, it is hard to ignore the capability of DNA testing to detect 97% of cases.” Not clear what "it is cost-effective compared to VIA alone" is referring to. If careHPV, should it be "less cost-effective compared to VIA alone" to align with the results? Please clarify.**

We have clarified that we are referring to VIA/VILI: “While our analysis indicates that VIA/VILI is cost-effective compared to VIA alone and *care*HPV, it is hard to ignore the capability of DNA testing to detect 97% of cases.”

**6. Line 459-460: "Loss to follow up circumvents the health gains that screening provides, consequently increasing the costs." Agree with the first part of this statement, however, LTFU may not necessarily result in increased costs when looking across the lifetime and evaluating cervical cancer cases averted (i.e., increased linkage to care often = higher costs associated with treatment). Suggest revising for clarity.**

We have revised this for clarity. It now reads: “Loss to follow up circumvents the health gains that screening provides, and a lifetime model would be needed to capture the costs and health outcomes associated with it.”
